# A Radiation Oncology Board Exam of ChatGPT

**DOI:** 10.7759/cureus.44541

**Published:** 2023-09-01

**Authors:** Andrew B Barbour, T. Aleksandr Barbour

**Affiliations:** 1 Radiation Oncology, University of Washington - Fred Hutchinson Cancer Center, Seattle, USA; 2 Starlink, SpaceX, Redmond, USA

**Keywords:** ai in medicine, ai tools, radiotherapy, radiation oncology, ai & robotics in healthcare

## Abstract

As artificial intelligence (AI) models improve and become widely integrated into healthcare systems, healthcare providers must understand the strengths and limitations of AI tools to realize the full spectrum of potential patient-care benefits. However, most providers have a poor understanding of AI, leading to distrust and poor adoption of this emerging technology. To bridge this divide, this editorial presents a novel view of ChatGPT’s current capabilities in the medical field of radiation oncology. By replicating the format of the oral qualification exam required for radiation oncology board certification, we demonstrate ChatGPT’s ability to analyze a commonly encountered patient case, make diagnostic decisions, and integrate information to generate treatment recommendations. Through this simulation, we highlight ChatGPT’s strengths and limitations in replicating human decision-making in clinical radiation oncology, while providing an accessible resource to educate radiation oncologists on the capabilities of AI chatbots.

## Editorial

Artificial intelligence (AI) includes a broad range of computational models, such as natural language processing (NLP), and has a variety of current and future applications in the healthcare setting [1]. In November 2022, the AI language model ChatGPT was publicly released (chat.openai.com) and quickly demonstrated proficiency in a diverse set of tasks, including United States Medical Licensing Examination questions [2]. ChatGPT was pre-trained on a large volume of text data and was designed to process and analyze human language; hence, ChatGPT is classified as an NLP and large language model. It is named GPT due to the design of Generative Pre-trained Transformer deep learning architecture, wherein a model is pre-trained on data, a transformer model neural network is used to process data, and human-like language is generated. When simulating human language, ChatGPT may provide convincingly false information, and its trustworthiness for use in radiation oncology has been questioned [3].

Most healthcare professionals have a poor understanding of AI, and providers are less likely to use AI in healthcare delivery if they do not trust or understand the technology [4,5]. To improve the understanding of AI by healthcare providers in radiation oncology, we interacted with ChatGPT version 3.5, asking a series of questions regarding the workup and management of a patient with unfavorable intermediate-risk prostate cancer. Below, we present a truncated version of this interaction, edited for brevity, and supplemented with author commentary for each response. This interaction included questions regarding diagnosis and staging (Table 1), treatment options (Table 2), and details of external-beam radiation treatment (Table 3).

**Table 1:** User input and ChatGPT output responses during the diagnosis and staging of a simulated patient with unfavorable intermediate-risk prostate cancer.

User input	ChatGPT output	Author commentary
You are a medical doctor. A 68-year-old man comes to your office with a PSA level of 11. What diagnostic tests would you like to order?	As a medical doctor, if a 68-year-old man comes to my office with a PSA level of 11 ng/mL, I would order further diagnostic tests to evaluate his prostate health and determine if he has prostate cancer. The following tests would be useful in making a diagnosis: DRE…, TRUS…, prostate biopsy…, MRI…, CT scan or bone scan… It is important to note that an elevated PSA level does not necessarily mean that a person has prostate cancer, as there are many other factors that can affect PSA levels. Therefore, the combination of several diagnostic tests is necessary to confirm a diagnosis	An extensive list of tests is provided, covering the main diagnostic tests traditionally used in the diagnosis and staging of prostate cancer. However, a prior history of PSA values, a family history of cancer, and a history that could provide non-oncologic reasons for PSA elevation should be obtained
You perform a DRE and the patient’s prostate is smooth without irregularity. A TRUS and prostate biopsy are performed. Of the 12 biopsy cores, 7 have prostate adenocarcinoma with a Gleason grade group of 3. MRI of the pelvis is obtained and is normal. Do you want any more information or diagnostic tests before providing a treatment recommendation?	Based on the information provided, a diagnosis of prostate cancer with a Gleason grade group of 3 has been made. The MRI of the pelvis is normal, which is a good sign that the cancer has not spread beyond the prostate gland. However, additional information would be helpful to guide treatment recommendations. The following tests or information could be considered: PSA velocity…, imaging of the prostate and surrounding tissues…, patient comorbidities… Based on this information, a multidisciplinary team consisting of a urologist, radiation oncologist, and medical oncologist can determine the best treatment plan for the patient. Treatment options may include surgery to remove the prostate gland, radiation therapy, or a combination of both. Additionally, the patient’s overall health status and personal preferences should be considered when making treatment recommendations	ChatGPT encounters difficulty with this question, providing answers that do not advance this patient’s management. At this stage, additional patient-specific information, such as urinary function and life expectancy, should be obtained, as well as imaging for detection of distant metastatic disease (e.g., NM bone scan)
The patient with a PSA of 11 has a normal rectal exam, a TRUS biopsy finding 7 out of 12 cores with Gleason grade group 3 prostate adenocarcinoma, a normal MRI of the pelvis, and a normal bone scan. What are the patient’s prostate cancer prognostic stage and risk group?	Based on the information provided, we can determine the patient’s prostate cancer prognostic stage and risk group using the American Joint Committee on Cancer (AJCC) staging system and the National Comprehensive Cancer Network (NCCN) risk classification system. Prognostic stage: The patient’s cancer is clinically classified as stage T1c (based on elevated PSA levels and biopsy findings), and there is no evidence of regional lymph node or distant metastasis on imaging studies. Therefore, the patient’s prostate cancer is categorized as stage II according to the AJCC staging system. Risk group: The NCCN risk group classification system is based on several factors, including PSA level, biopsy findings, and clinical stage. Based on the information provided, the patient’s NCCN risk group is intermediate risk… The patient has a PSA level of 11 ng/mL and biopsy findings of 7 out of 12 cores with Gleason grade group 3 (which is equivalent to Gleason score 7), which places him in the intermediate-risk group	An appropriate answer is provided, correctly classifying the patient as intermediate risk
Is the patient’s risk group favorable intermediate or unfavorable intermediate?	The patient has a PSA level of 11 ng/mL and biopsy findings of 7 out of 12 cores with Gleason grade group 3 (which is equivalent to Gleason score 7), which places him in the intermediate-risk group. To determine if the patient’s risk group is favorable or unfavorable intermediate, we need to consider additional factors, such as age…, PSA velocity…, percent of biopsy cores involved…, clinical stage… Without additional information, it is difficult to definitively classify the patient’s risk group as favorable or unfavorable intermediate. However, based on the information provided, the patient’s risk group is likely closer to favorable intermediate, as his PSA level is relatively low, and the cancer appears to be confined to the prostate gland on imaging studies. However, the patient’s overall health status and personal preferences should also be considered when making treatment recommendations. It is important for the patient to discuss treatment options with a multidisciplinary team of healthcare professionals to determine the most appropriate treatment plan	An incorrect answer is provided. This patient is unfavorable intermediate risk given his two intermediate-risk factors (PSA >10 and grade group 3). Having >50% of biopsy cores positive could also be used to classify this patient’s risk group

**Table 2:** User input and ChatGPT output responses regarding the treatment options of a simulated patient with unfavorable intermediate-risk prostate cancer.

User input	ChatGPT output	Author commentary
You are a urologist. What treatment options would you provide to this patient?	As a urologist, there are several treatment options that I would discuss with this patient, which may include: (1) Active surveillance: For some patients with low-risk or favorable intermediate-risk prostate cancer, active surveillance may be a reasonable option. This involves monitoring the cancer with regular PSA tests, digital rectal exams, and repeat biopsies, and delaying treatment until the cancer shows signs of progression. (2) Surgery: Radical prostatectomy, which involves removing the entire prostate gland, may be recommended for patients with intermediate-risk prostate cancer. This treatment is typically recommended for patients with a life expectancy of greater than 10 years and who have a life expectancy that is longer than the time required for recovery from surgery. (3) Radiation therapy: External beam radiation therapy (EBRT) or brachytherapy (implantation of radioactive seeds into the prostate gland) may be recommended for patients with intermediate-risk prostate cancer… (4) Combination therapy: For some intermediate-risk prostate cancer patients, a combination of surgery and radiation therapy may be recommended to increase the chance of curing the cancer. (5) Androgen deprivation therapy (ADT): ADT is a form of hormone therapy that reduces the levels of male hormones (androgens), which can cause prostate cancer cells to grow. ADT may be used in combination with radiation therapy for intermediate-risk prostate cancer patients	Two appropriate treatment options are discussed, including prostatectomy and combination therapy with radiation and ADT. However, the other treatment options provided are not compatible with current management guidelines and represent broad management options that are not applicable to this patient
You are a radiation oncologist and see this patient in your office. The patient has unfavorable intermediate-risk prostate cancer and has completed his staging work-up, showing no evidence of prostate cancer outside of the prostate. The patient would like combination therapy with EBRT and ADT. What radiation dose and fractionation would you recommend?	For patients with unfavorable intermediate-risk prostate cancer who receive EBRT and ADT, a radiation dose of 78 Gy in 39 fractions over approximately 8 weeks is the standard of care based on results from multiple randomized clinical trials. The use of ADT in combination with radiation therapy has been shown to improve disease-free survival and overall survival in patients with intermediate and high-risk prostate cancer. The radiation dose and fractionation can be adjusted depending on several factors, including the patient’s age, overall health status, and treatment-related toxicities. In some cases, a higher radiation dose of up to 80 Gy in 40 fractions may be considered in patients with higher-risk disease, although this can increase the risk of side effects such as urinary and bowel toxicity. It is important to discuss the potential benefits and risks of radiation therapy and ADT with the patient, including potential side effects such as fatigue, erectile dysfunction, and hot flashes. Additionally, the patient's overall health status, personal preferences, and the availability of radiation therapy and ADT resources should be considered when developing an individualized treatment plan	A dose and fractionation is provided that is allowable per the NCCN guidelines, but is neither favored nor broadly used in current practice
How many months of androgen deprivation therapy would you recommend?	The duration of androgen deprivation therapy (ADT) in combination with radiation therapy for intermediate-risk prostate cancer is a topic of ongoing research and debate. The standard duration of ADT in combination with radiation therapy is currently 18–36 months based on the results of several clinical trials, although some studies have suggested that longer durations of ADT may provide additional benefits. For patients with unfavorable intermediate-risk prostate cancer, a duration of 24–36 months of ADT is often recommended in combination with radiation therapy. However, the optimal duration of ADT should be individualized based on the patient's risk profile, age, overall health status, and personal preferences. It is important to discuss the potential benefits and risks of ADT with the patient, including potential side effects such as hot flashes, decreased libido, erectile dysfunction, and osteoporosis. Additionally, the patient’s overall health status, personal preferences, and the availability of ADT resources should be considered when developing an individualized treatment plan	An incorrect answer is provided. The provided answer is applicable to patients with high-risk disease, whereas this patient should be recommended 4–6 months of ADT

**Table 3:** User input and ChatGPT output responses regarding external beam radiation treatment of a simulated patient with unfavorable intermediate-risk prostate cancer.

User input	ChatGPT output	Author commentary
When treating this unfavorable intermediate-risk prostate adenocarcinoma patient with EBRT, how would you design your treatment volume?	When designing the treatment volume for external beam radiation therapy (EBRT) for unfavorable intermediate-risk prostate adenocarcinoma, a combination of pelvic and prostate-only radiation fields is typically used. The pelvic radiation field is used to treat the pelvic lymph nodes and may include the external iliac, internal iliac, and obturator lymph node regions. The prostate-only radiation field is used to treat the prostate gland itself. The pelvic radiation field is typically used for patients with intermediate- or high-risk prostate cancer, as there is a risk of microscopic spread of the cancer cells to the pelvic lymph nodes. However, the use of pelvic radiation can increase the risk of side effects such as bowel and bladder toxicity. The prostate-only radiation field may be used for patients with low-risk prostate cancer, as the risk of spread to the pelvic lymph nodes is low. However, for patients with unfavorable intermediate-risk prostate cancer, a combination of pelvic and prostate-only radiation fields is typically used to reduce the risk of recurrence. The specific design of the radiation fields will depend on the patient’s individual anatomy and the location of the prostate gland relative to the pelvic lymph nodes. A CT scan or MRI may be used to help guide the radiation planning process and ensure that the treatment volume is accurately targeted while minimizing radiation exposure to surrounding healthy tissue	In fairness to ChatGPT, there are differences in clinical practice in this scenario. Most providers treat the prostate alone in the modern era, although some still would recommend whole pelvic radiation in this setting
You decide to treat this patient’s prostate to 78 Gy in 39 fractions and decide to also use a pelvic radiation field to treat the pelvic lymph nodes. What dose and fractionation do you prescribe to the pelvic radiation field?	When treating the pelvic lymph nodes in combination with prostate radiation therapy for unfavorable intermediate-risk prostate cancer, the standard dose and fractionation is 45 Gy in 25 fractions over approximately 5 weeks. However, the optimal dose and fractionation for pelvic radiation therapy in this setting is still an area of active research and may depend on various factors such as the patient’s individual risk profile, overall health status, and the potential for treatment-related side effects…	An appropriate dose for sequential elective nodal irradiation is provided
What radiation treatment technique would you use when treating this patient with EBRT?	When treating a patient with external beam radiation therapy (EBRT) for prostate cancer, several radiation treatment techniques can be used, including three-dimensional conformal radiation therapy (3D-CRT), intensity-modulated radiation therapy (IMRT), and volumetric-modulated arc therapy (VMAT). IMRT and VMAT are advanced radiation techniques that allow for the delivery of high doses of radiation to the prostate while minimizing radiation exposure to surrounding healthy tissue. These techniques use computer-controlled linear accelerators to deliver radiation beams that are shaped to conform to the shape of the prostate gland and spare nearby organs such as the bladder and rectum. IMRT uses multiple radiation beams that are delivered at different intensities and angles, while VMAT uses a single radiation beam that rotates around the patient, allowing for more precise and efficient delivery of radiation… In this case, either IMRT or VMAT could be used to deliver the prescribed radiation dose to the prostate and pelvic lymph nodes while minimizing radiation exposure to surrounding healthy tissue	An appropriate answer is provided, and extraneous details describing these techniques is provided
When designing EBRT to the prostate and pelvic lymph nodes for this unfavorable intermediate-risk prostate adenocarcinoma patient, what are the most important organs at risk to consider? What dosimetric constraints would you place on these organs at risk?	When designing external beam radiation therapy (EBRT) for the prostate and pelvic lymph nodes, there are several organs at risk that need to be carefully considered. The most important organs at risk include the rectum, bladder, and femoral heads. For the rectum, the dosimetric constraints typically include limiting the maximum dose to less than 75 Gy and limiting the volume of the rectum receiving more than 70 Gy (V70) to less than 15%. For the bladder, the constraints may include limiting the maximum dose to less than 80 Gy and limiting the volume of the bladder receiving more than 65 Gy (V65) to less than 30%. For the femoral heads, the constraints may include limiting the mean dose to less than 45 Gy and limiting the volume of the femoral heads receiving more than 50 Gy (V50) to less than 50%…	Three major organs at risk are identified. While most dose constraints are technically appropriate, the femoral head V50 is unacceptably high in this setting. It appears ChatGPT selects a constraint used in a different disease setting (soft tissue sarcoma), where higher femoral head doses are allowed
You begin treating this patient with external beam radiation. After five weeks of treatment, the patient describes feeling like it is more difficult to initiate urination and describes a weaker urinary stream. How do you manage this complaint?	Difficulty initiating urination and a weaker urinary stream are common side effects of radiation therapy to the prostate. Here are some steps to manage this complaint: (1) Evaluate the patient: The first step is to evaluate the patient to determine the cause of the urinary symptoms. A physical exam, including a digital rectal exam, can help assess the size and consistency of the prostate. A bladder scan or uroflowmetry can help evaluate the patient's urinary function. (2) Manage symptoms: If the symptoms are due to radiation-induced inflammation, supportive measures such as increased hydration, a bladder relaxant medication, and/or alpha-blocker medication can be used to help manage symptoms. These medications can help relax the muscles around the prostate and improve urinary flow. (3) Consider referral to a urologist: If symptoms persist or worsen despite supportive measures, a referral to a urologist may be necessary. The urologist can evaluate the patient's urinary function and may recommend further treatment such as a urethral dilation or medication to improve urinary function	A reasonable overall approach is provided. However, a digital rectal exam may not be needed in this setting, instead, a patient history of symptoms and current medication use should be obtained
How much will this patient’s radiation treatment cost in US dollars?	The cost of radiation treatment for prostate cancer can vary widely depending on many factors, including the geographic location of the treatment center, the type of radiation therapy used, and the specifics of the individual patient’s treatment plan. Other factors that may impact the cost of treatment include the duration of treatment, the need for additional imaging studies, and any additional medications or procedures required to manage side effects. It is difficult to estimate the cost of this patient’s radiation treatment without more specific information about the treatment plan and location. I would recommend contacting the treatment center or the patient’s insurance provider for more specific information about the cost of treatment	ChatGPT provides an answer that is surprisingly similar to what most healthcare practitioners would offer

In conclusion, ChatGPT-3.5 was able to provide algorithmic information but was limited by verbosity and incomplete synthesis of complex information. Throughout the interaction, ChatGPT did not pose a single clarifying question to the author. In the initial workup, ChatGPT failed to take an initial patient history and neglected to elucidate the urinary, bowel, and sexual function of the patient. The diagnostic approach was overly broad and would lead to overtesting in practical application. While ChatGPT collected the necessary elements required for staging the patient, it was unable to correctly identify the patient as having unfavorable intermediate-risk prostate cancer, despite specific prompting. ChatGPT presented an exhaustive list of options for the management of organ-confined prostate cancer, including several that were not appropriate for the clinical situation. The specifics of radiation dose fractionation, field design, and treatment delivery were accurate, but a largely antiquated dose was prescribed, an excessive duration of androgen deprivation therapy was recommended, and an incorrect dosimetric constraint for the femoral heads was provided. Although the cutoff date for ChatGPT-3.5 training data was in 2021, all information needed to answer the prompted questions existed before this date. While ChatGPT passed some portions of the examination, its overall performance was inadequate, highlighting the importance of trained human decision-making. However, the shortcomings must be taken in the context of ChatGPT’s generalized training to simulate human language (as opposed to conducting medical tasks), sensitivity to prompt engineering, and improvements in GPT-4 that may address verbosity.
